# OCCUPATIONAL HEALTH: Study to Examine Health Effects in Deepwater Horizon Oil Spill Cleanup Workers

**DOI:** 10.1289/ehp.119-a204

**Published:** 2011-05

**Authors:** Charles W. Schmidt

**Affiliations:** **Charles W. Schmidt**, MS, an award-winning science writer from Portland, ME, has written for *Discover Magazine*, *Science*, and *Nature Medicine*

In the wake of the BP *Deepwater Horizon* disaster in the Gulf of Mexico, the National Institute of Environmental Health Sciences (NIEHS) has launched the largest study ever on the health consequences of oil spill cleanup.^1^ Led by Dale Sandler, who heads the NIEHS’s intramural Epidemiology Branch, the GuLF STUDY (Gulf Long-term Follow-up study) aims to enroll 55,000 workers and volunteers involved in the BP oil spill response, including 5,000 controls.^2^

National Institutes of Health director Francis Collins pledged $10 million for the study on 15 June 2010.^3^ Sandler says the full cohort should be assembled within 18–24 months. “We’ve got funding for five years, but we designed the study so it can go up to twenty years,” she says. “If we want to assess links between oil spill response and rare cancers, we’ll have to go that far.”

Nalini Sathiakumar, an environmental and occupational epidemiologist at the University of Alabama at Birmingham School of Public Health, says the GuLF STUDY’s size and prospective design distinguish it from earlier health investigations of oil spill responses. “Previous studies primarily examined short-term health effects and were cross-sectional, meaning they looked at exposure outcomes at just one time point,” she says. “What you really want to do is a longitudinal assessment of exposure–outcome relationships, and that’s what this study does.”

According to Sandler, the GuLF STUDY will focus especially on respiratory, neurological, and hematological outcomes linked in the toxicologic literature to oil constituents. And taking a cue from other environmental disasters such as the nuclear incident at Three Mile Island and the World Trade Center attacks, she says, where many people reported mental stress,^4^,^5^ the GuLF STUDY will also investigate psychological outcomes in relation to cleanup and living in the Gulf region.

Exposure to volatile organic compounds (VOCs), polycyclic aromatic hydrocarbons (PAHs), and dispersants will be estimated based partly on environmental monitoring data gathered by agencies and organizations during the response, Sandler says. Those data will be used to construct job-exposure matrices, which will also be informed by self-reported answers to a telephone questionnaire the NIEHS began administering in late March. The questionnaire requests information about where individuals worked during the cleanup, how long they worked, what they did, and whether they used personal protective equipment, in addition to background information about family health history, lifestyle, diet, mental health status, and prior employment history.

In addition, investigators will collect samples of blood, hair, toenail, urine, and other biospecimens from about half the participants to search for biomarkers showing some evidence of interaction with or influence on a biological process. DNA adducts, chromosome damage, and altered ability to repair DNA are examples of the sorts of biomarkers being considered.

Ideally, the study will allow scientists to link job categories and estimated exposures during the cleanup to a range of frank and subtle health effects over time, Sandler says. The NIEHS will publicize results in periodic summary reports, “and anyone who wants access to the data will be able to submit a request,” Sandler says. “Scientists will have to honor confidentiality—it’s important to respect the privacy of data given to us in confidence.”

Bill Farland, senior vice president for research at Colorado State University in Fort Collins and an invited speaker at a June 2010 Institute of Medicine workshop titled “Assessing the Human Health Effects of the Gulf of Mexico Oil Spill,” describes the study as well designed, particularly because it investigates both physiological and mental health outcomes. “What we’re most concerned about are the VOC and PAH exposures,” he says. “This is what people who were exposed to the oil slick itself would have come in contact with.”

## Figures and Tables

**Figure:**
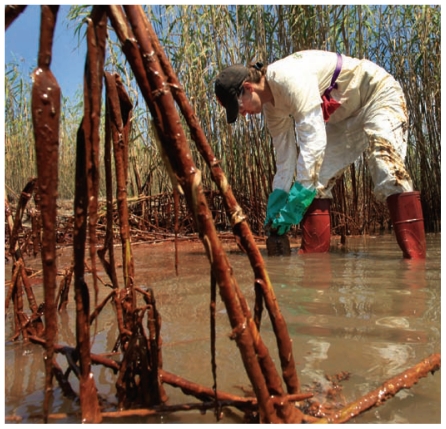
A new NIEHS study will examine health outcomes in oil spill workers.
